# Similar cortical morphometry trajectories from 5 to 9 years in children with perinatal HIV who started treatment before age 2 years and uninfected controls

**DOI:** 10.1186/s12868-023-00783-7

**Published:** 2023-02-24

**Authors:** Emmanuel C. Nwosu, Martha J. Holmes, Mark F. Cotton, Els Dobbels, Francesca Little, Barbara Laughton, Andre van der Kouwe, Frances Robertson, Ernesta M. Meintjes

**Affiliations:** 1 Biomedical Engineering Research Centre, Division of Biomedical Engineering, Department of Human Biology, Faculty of Health Sciences, University of Cape Town, Anzio Road, Observatory, Cape Town, 7925 South Africa; 2 Neuroscience Institute, University of Cape Town, Cape Town, South Africa; 3 Department of Pediatrics & Child Health, Family Centre for Research With Ubuntu (FAMCRU), Tygerberg Hospital, Stellenbosch University, Cape Town, South Africa; 4 Department of Statistical Sciences, University of Cape Town, Cape Town, South Africa; 5 A.A. Martinos Centre for Biomedical Imaging, Massachusetts General Hospital, Boston, MA USA; 6 Department of Radiology, Harvard Medical School, Boston, MA USA; 7 Cape Universities Body Imaging Centre, University of Cape Town, Cape Town, South Africa

**Keywords:** Longitudinal, Children with HIV, CHER, Cortical thickness, Gyrification, FreeSurfer, Vertex-wise

## Abstract

**Background:**

Life-long early ART (started before age 2 years), often with periods of treatment interruption, is now the standard of care in pediatric HIV infection. Although cross-sectional studies have investigated HIV-related differences in cortical morphology in the setting of early ART and ART interruption, the long-term impact on cortical developmental trajectories is unclear. This study compares the longitudinal trajectories of cortical thickness and folding (gyrification) from age 5 to 9 years in a subset of children perinatally infected with HIV (CPHIV) from the Children with HIV Early antiRetroviral therapy (CHER) trial to age-matched children without HIV infection.

**Methods:**

75 CHER participants in follow-up care at FAMCRU (Family Centre for Research with Ubuntu), as well as 66 age-matched controls, received magnetic resonance imaging (MRI) on a 3 T Siemens Allegra at ages 5, 7 and/or 9 years. MR images were processed, and cortical surfaces reconstructed using the *FreeSurfer* longitudinal processing stream. Vertex-wise linear mixed effects (LME) analyses were performed across the whole brain to compare the means and linear rates of change of cortical thickness and gyrification from 5 to 9 years between CPHIV and controls, as well as to examine effects of ART interruption.

**Results:**

Children without HIV demonstrated generalized cortical thinning from 5 to 9 years, with the rate of thinning varying by region, as well as regional age-related gyrification increases. Overall, the means and developmental trajectories of cortical thickness and gyrification were similar in CPHIV. However, at an uncorrected *p* < 0.005, 6 regions were identified where the cortex of CPHIV was thicker than in uninfected children, namely bilateral insula, left supramarginal, lateral orbitofrontal and superior temporal, and right medial superior frontal regions. Planned ART interruption did not affect development of cortical morphometry.

**Conclusions:**

Although our results suggest that normal development of cortical morphometry between the ages of 5 and 9 years is preserved in CPHIV who started ART early, these findings require further confirmation with longitudinal follow-up through the vulnerable adolescent period.

**Supplementary Information:**

The online version contains supplementary material available at 10.1186/s12868-023-00783-7.

## Background

As of mid-2021, there were an estimated 1.3–2.1 million children with perinatal HIV infection (CPHIV) under 15 years of age living in sub-Saharan Africa (SSA) [1]. Although the number of new vertical infections is declining due to implementation of strategies for prevention of vertical transmission, there are still about 310,000 children (age ≤ 14 years) living with HIV in South Africa [1]. Early antiretroviral therapy (ART) initiation upon testing HIV seropositive is the current standard of care for CPHIV [2–4]. However, the consequences of long-term ART on the developing brain are not well understood. As such, there is a need for longitudinal studies of brain development in well-described cohorts of CPHIV in the context of early ART.

A key study that led to the global adoption of early ART was the children with HIV early antiretroviral therapy (CHER) trial, which was conducted between 2005 and 2011 in Johannesburg and Cape Town, South Africa. The study aimed to investigate clinical outcomes of early (age ≤ 12 weeks) time-limited ART in asymptomatic infants with perinatal HIV (PHIV) compared to deferred, continuous ART. Clinical, neuropsychological and neuroimaging follow-up of the Cape Town CHER participants was performed to investigate the long-term effects of perinatal infection and early ART initiation on the central nervous system (CNS), which is a key viral reservoir, even in the combination ART (cART) era [5].

In this cohort, both neuropsychological testing [6–8] and cross-sectional neuroimaging investigations suggest small but persistent effects of HIV on the developing brain, despite viral suppression from a young age. In subcortical regions, our group has found HIV-related volume increases and decreases at different ages [9–11]. At 5- and 9 years HIV-related differences in metabolite levels in the basal ganglia (BG) were observed [12, 13], and at 11 years in the midfrontal gray matter (MFGM) and peritrigonal white matter (PWM) [14]. In terms of white matter development, at 5- and 7 years we found HIV-associated abnormalities in localized white matter integrity [15, 16]. Even though no HIV-related microstructural differences were evident in the corpus callosum at these ages, corpus callosum volumes were smaller in children with PHIV at age 5 [9].

Looking at cortical gray matter, at 5 years we found HIV-associated cortical thickness (CT) increases in bilateral frontal and left temporal regions, as well as reductions in local gyrification indices (LGI) in bilateral medial orbitofrontal regions extending into the rostral anterior cingulate and left superior frontal regions [11]. At 7 years, CPHIV demonstrated thicker cortex in a small left lateral occipital region and less gyrification in bilateral paracentral and right temporal regions [10].

ART interruption could exacerbate the ongoing impact of HIV on brain development in CPHIV. Several circumstances could lead to ART interruption including, intolerance of ART in children since pediatric formulation and pill sizes are not readily available, stock-outs and, more recently, the test for HIV remission which requires intensive monitored antiretroviral pause [17–20]. Lewis et al. [21] indicated that while ART interruption may have limited effect on children in the short term, its impact may be significant during critical periods of neural development. We previously identified region specific differences in cortical thickness and folding in relation to ART interruption at age 5 years in a sub-group of the CHER cohort using a cross-sectional approach but have not investigated further the long-term impact of interruption using a longitudinal approach [11].

While HIV and ART interruption-related alterations have been observed cross-sectionally at ages 5, 7 and/or 9 years, many of the alterations are not observed across multiple time points. Longitudinal analysis across these ages (5–9 years) may clarify the longer-term consequences of cross-sectional findings. Longitudinal data are informative because they allow the modeling of developmental changes as either physiologically normal or pathological in relation to various disease conditions [22], thereby enhancing understanding of developmental patterns in the presence of these conditions. Longitudinal neuroimaging studies are increasingly desirable in neuroimaging research due to their high sensitivity and potential to account for inter-subject variability [23]. For example, our recent longitudinal analysis of metabolite levels in children from the CHER trial revealed elevated choline levels indicative of neuroinflammation in CPHIV compared to uninfected controls from 5–11 years in all three regions studied, namely BG, MFGM and PWM [24].

Brain morphometric measures such as CT—the distance between the inner white/gray matter boundary and the outer gray/pial interface in the cerebral cortex [25–27] and LGI—a measure of cortical folding, which increases the number of neurons and cortical surface area within a limited skull space [22, 28, 29]—can provide valuable information on cortical anatomical development. Important micro-anatomical changes, including synaptic pruning, neuronal specialization, rewiring of fibre tracts, and structural folding and compacting occur during development from late childhood into adolescence, and determine the subsequent optimal function of the brain in adult life [22, 30–32]. Longitudinal investigation of these microstructural parameters in children living with HIV is key to understanding whether the developmental trajectory of cortical anatomy during a critical period of brain development [22, 30–32] differs from that of uninfected controls.

The aim of this study was therefore to describe the longitudinal trajectory of morphometric development of the cerebral cortex between the ages of 5 and 9 years in the Cape Town arm of the CHER cohort, all of whom started ART before 2 years of age. We hypothesized that early-treated CPHIV would demonstrate altered morphometric development compared to uninfected controls in regions where we previously observed HIV-related differences on cross-sectional analyses. In addition, based on effects of ART interruption seen at age 5 years, we posited that changes in cortical thickness development would only be evident in CPHIV in whom ART had been interrupted, while LGI development would be altered similarly in all CPHIV (irrespective of whether treatment had been interrupted or not).

## Methods

### Study participants

Study participants were 141 children (75 with PHIV; 72 male) who are being followed longitudinally at the Family Centre for Research with Ubuntu (FAMCRU) at Tygerberg Children’s Hospital in Cape Town, South Africa. The CPHIV were from the Cape Town cohort of the CHER clinical trial on which infants with CD4 percentage ≥ 25% were randomized at age 6 -12 weeks to receive either immediate limited ART—interrupted at 40 or 96 weeks—and restarted when clinical and/or immunological criteria were met, or to start ART only when they developed HIV symptoms or CD4 percentage dropped below 20% (25% in the first year) as per guidelines at the time [33]. Enrolled children received comprehensive immunological and clinical follow-up, including assessment for HIV-related encephalopathy. All CPHIV in the present sample started ART before 76 weeks of age. First line ART regimen consisted of Zidovudine (ZDV) + Lamivudine (3TC) + Lopinavir-Ritonavir (LPV/r, Kaletra®) [2, 34]; all CPHIV were on first line ART regimen until the end point of the clinical trial [2]. Socioeconomically- and sociodemographically matched children without HIV infection (HIV-) were recruited from an interlinking vaccine trial [35]. The uninfected control group comprised both children born to mothers with HIV infection (children who are HIV exposed uninfected, CHEU; n = 31) and to HIV- mothers (children who are HIV unexposed; CHU; n = 35). To increase power to detect HIV-related differences, we combined CHEU and CHU groups in our analyses. *Posthoc* analyses were performed in any regions showing HIV-related differences in morphometric development to confirm whether exposed and unexposed children differed in those regions.

While CPHIV were motivated to remain in the longitudinal study as they received clinical care and neuropsychological assessments, there was limited incentive for children in the control group to remain in the study. This resulted in higher attrition rates among the control group. To combat this, additional controls were recruited during the study from similar neighbourhoods as the CHER children. As far as possible, newly recruited controls were matched with respect to exposure status to those lost to follow-up (LTFU). Eligibility criteria included documented HIV status of the mother and the child at birth, birthweight > 2000 g, being clinically healthy with normal medical history, no history of CNS insults or birth complications, nor a medical or psychiatric disorder that might affect cognitive performance [6, 36]. The current sample includes 24 additional controls (6 CHEU, 18 CHU) who were recruited at age 7 years.

### Image acquisition and analysis

Study participants underwent brain MRI scanning on a Siemens 3 T Allegra scanner at 5, 7 and 9 years of age, using a protocol and procedure described previously [10]. Scans were performed without sedation according to protocols approved by the Faculty of Health Sciences Human Research Ethics Committees of the Universities of Cape Town and Stellenbosch. Parents/guardians of study participants provided written informed consent and the children gave oral assent at ages 5 and 7, and written assent at 9 years. To limit motion due to restlessness, children watched a movie via a mirror and rear projection screen during scanning. Furthermore, we used a 3D echo planar imaging (EPI) navigated [37] multiecho magnetization prepared rapid gradient echo (MEMPRAGE) [38] sequence (FOV 224 × 224 mm2, TR 2530 ms, TI 1160 ms, TEs = 1.53/3.19/4.86/6.53 ms, bandwidth 657 Hz/px, 144 slices, 1.3 × 1.0 × 1.0 mm3) that prospectively corrects for motion during the scan. MR images were visually inspected and those with poor visual quality were excluded from analyses.

MR images were reviewed by a senior radiologist; if abnormalities were noted, children were referred for a clinical scan. T1-weighted high-resolution structural volumes that met visual quality control criteria were processed using the automated 3-stage longitudinal processing stream in FreeSurfer version 6.0 to extract reliable longitudinal surface CT and LGI measures (https://surfer.nmr.mgh.harvard.edu/fswiki/LongitudinalProcessing). The longitudinal processing stream first performs cross-sectional parcellation and cortical surface reconstruction for all subjects at all time points. The next stage of the pipeline re-samples the cross-sectional data of each subject to a base template. From the base template, longitudinal data are generated at all time points [23, 39, 40]. Outputs from the segmentation and parcellation steps were visually checked for errors and outlier values for CT/LGI were identified. Reconstructed data were then sampled to the FreeSurfer average subject template for vertex-wise analysis. For CT analyses, spatial smoothing was applied using a Gaussian kernel with 10 mm full-width, half-maximum (FWHM). No smoothing was used for LGI.

### Longitudinal vertex-wise analyses of cortical thickness and LGI

Morphometric data were analysed using FreeSurfer’s spatiotemporal linear mixed effect model (LME) toolbox in MATLAB R2017a (https://www.mathworks.com/) [41]. LME models can handle longitudinal data such as ours that contains observations at different time points and varying numbers of time points for different subjects. To establish regional CT and LGI trajectories in typically developing children, we first created an LME model with HIV- children only. To investigate the influence of HIV in the context of early treatment on morphometric development across this age range, we performed an analysis on all participants using an LME model that additionally included both HIV status and an age by HIV status interaction term to identify possible HIV-related differences in developmental trajectories.

Further, the rates of change in CT and LGI of CPHIV who had interrupted ART (Interrupted ART) and those who had continuous ART (Continuous ART) were separately compared to HIV- controls. To assess the possible effect of encephalopathy on the trajectory of cortical development in PHIV, we also compared children who had previously had a diagnosis of HIVE to HIV- controls.

All analyses were done using a vertex-wise LME model, with group as a categorical fixed effect and random participant-specific intercepts. Rate of CT and LGI change with age in HIV- control children were modelled as follows:

1 $${\text{Y}}_{{{\text{ij}}}} = \,\beta_{{1}} + \, \left( {\beta_{{2}} *{\text{ t}}_{{{\text{ij}}}} } \right) \, + \, \left( {\beta_{{3}} *{\text{ sex}}_{{\text{i}}} } \right) \, + {\text{ b}}_{{{\text{1i}}}} + {\text{e}}_{{{\text{ij}}}}$$

where Y_*ij*_ = cortical thickness or gyrification of subject i at time point *j* β_1_ = intercept t_*ij*_ = time variable—age in years of subject *i* at scan time point *j* sex_i_ = 1 if subject *i* was female and 0 if male

b_1i_ = participant-specific intercept

e_ij_ = error

To examine potential non-linear effects of time, we repeated our analyses with an additional term for the square of time (t_*ij*_^2^) added in the model. For both CT and LGI, we present colour maps of the regression coefficients for age (i.e. β_2_ from Eq. 1) across the whole cortical surface.

To allow for different means or slopes in PHIV (or Interrupted ART or Continuous ART or HIVE) and HIV- children, the following model was used:

2 $${\text{Y}}_{{{\text{ij}}}} = \, \beta_{{1}} + \, \left( {\beta_{{2}} *{\text{ t}}_{{{\text{ij}}}} } \right) \, + \, \left( {\beta_{{3}} *{\text{ HIV}}_{{\text{i}}} } \right) \, + \left( {\beta_{{4}} *{\text{ HIV}}_{{\text{i}}} *{\text{ t}}_{{{\text{ij}}}} } \right) + \, \left( {\beta_{{5}} *{\text{ sex}}_{{\text{i}}} } \right) \, + {\text{ b}}_{{{\text{1i}}}} + {\text{e}}_{{{\text{ij}}}}$$

where

HIV_i_ = variable for HIV status of subject *i* (or HIVE status or Interrupted ART or Continuous ART).

The null hypothesis of differences in either the means or rate of change of CT/LGI between PHIV (or Interrupted ART or Continuous ART or HIVE) and HIV- groups was tested. We also investigated potential non-linear effects of time by repeating the analyses with a term for the square of time (t_*ij*_^2^) added into the model. To correct for multiple comparisons we used a false discovery rate (FDR) corrected threshold of *p* < 0.05 for a two-tailed test. For both CT and LGI, we present colour maps of the relevant regression coefficients (i.e. β_3_ and β_4_ from Eq. 2 for group and age by group interaction effects, respectively) across the entire cortical surface.

## Results

We present results for 141 participants who received MRI at least once from 5 to 9 years on the same scanner. Fifty-six children (23 PHIV) received a single scan, 63 children (44 PHIV) were scanned twice, and 22 children (8 PHIV) had 3 scans, for a total of 248 time points. Viral loads of 93.5% of the children in this study were suppressed (< 400 copies/mL) at the first scan around age 5 years. The 5-year data of one HIV- control child were excluded due to being incomplete, and the data at age 7 years of one CPHIV were excluded due to poor image quality. There were no failures in automated LGI computation, nor errors in cortical segmentation. There were no outliers in output values of CT and LGI for processed MR images. Table 1 shows biographical data of children without (HIV-) and with PHIV at ages 5, 7 and 9 years. Table 2 presents clinical data for the CPHIV.

**Table 1:** Biographical data for all participants (N = 141; 75 PHIV, 66 HIV-)

	CPHIV			HIV-			*t*/χ^2^		*p*
**Age: 5 years (N = 82)**									
Sample size (N)	52			30					
Age at scan (years)	5.39 ± 0.31			5.60 ± 0.43			− 2.64		**0.01**
Number of males (% males)	21 (40%)			16 (53%)			1.37		0.24
Birth weight (g)	3125 ± 386			2973 ± 603			1.47		0.14
Estimated total intracranial volume (ETIV) (cm^3^)	1375 ± 96			1363 ± 110			0.53		0.60
Neuropsychological measures GMDS^a^	All CPHIV	IntART (n = 24)	ConART (n = 28)	All HIV-	CHEU (n = 17)	CHU (n = 13)			
Performance (EQ)	74 ± 10	73 ± 11	76 ± 10	76 ± 18	73 ± 20	79 ± 17	− 0.55		0.58
Practical reasoning (FQ)	77 ± 8	76 ± 10	77 ± 7	76 ± 11	73 ± 12	79 ± 7	0.60		0.55
Sub-scales aggregate (GQ)	83 ± 6	83 ± 7	83 ± 6	83 ± 8	82 ± 10	84 ± 6	0.23		0.82
**Age: 7 years (N = 125)**									
Sample size (N)	71			54					
Age at scan (years)	7.20 ± 0.13			7.24 ± 0.13			− 1.40		0.16
Number of males (% males)	36 (51%)			30 (56%)			0.13		0.72
Birth weight (g)	3081 ± 454			3081 ± 481			− 0.01		0.99
Estimated total intracranial volume (ETIV) (cm^3^)	1419 ± 101			1444 ± 137			− 1.17		0.24
Neuropsychological measures KABC^b^ COMPOSITE SCORES	All CPHIV	IntART (n = 32)	ConART (n = 39)	All HIV-	CHEU (n = 23)	CHU (n = 31)			
Mental processing index	72 ± 8	72 ± 8	73 ± 8	75 ± 10	74 ± 10	76 ± 11	− 1.90		0.06
Non-verbal index	74 ± 11	73 ± 11	75 ± 11	76 ± 12	73 ± 12	78 ± 11	− 0.96		0.34
**Age: 9 years (N = 41)**									
Sample size (N)	12			29					
Age at scan (years)	9.13 ± 0.27			9.03 ± 0.56			0.58		0.57
Number of males (% males)	7 (58%)			16 (55%)			< 0.001		1.00
Birth weight (g)	3126 ± 479			3171 ± 466			− 0.28		0.78
Estimated total intracranial volume (ETIV) (cm^3^)	1422 ± 683			1437 ± 135			− 0.38		0.71
Neuropsychological measures KABC^b^ COMPOSITE SCORES	All CPHIV	IntART (n = 8)	ConART (n = 4)	All HIV-	CHEU (n = 17)	CHU (n = 12)			
Mental processing index	73 ± 10	73 ± 10	72 ± 10	79 ± 12	77 ± 12	81 ± 12	− 1.49		0.14
Non-verbal index	78 ± 10	78 ± 8	77 ± 15	80 ± 14	80 ± 14	80 ± 13	− 0.46		0.65

All values are mean ± standard deviation except where indicated otherwise; Bold indicates significance at *p* < 0.05

*CPHIV* children with perinatal HIV; HIV- children without HIV, *IntART* children with PHIV in whom ART was interrupted; *ConART* children with PHIV who received continuous ART; *CHEU* uninfected children who were exposed to HIV; *CHU* uninfected children unexposed to HIV

^a^Griffiths’ Mental Development Scales—extended revised: 2–8 years

^b^Kaufman Assessment Battery for Children, second edition

**Table 2:** Clinical data for all children with PHIV (N = 75)

	**Interrupted ART**	**Continuous ART**
Sample size (N)	39	36
***Clinical data at baseline (age 6–8 weeks)***		
CD4 count (cells/mm^3^)	1827 ± 955	1739 ± 833
CD4%	33 ± 10	33 ± 10
CD4/CD8 ratio	1.4 ± 0.9 [0.2–4.4]^a^	1.2 ± 0.7 [0.2–3.4]^a^
CD8 count (cells/mm^3^)	1660 ± 1218	1686 ± 871
Viral load at enrolment		
High (>750,000 copies/mL): n (%)	21 (53.8%)	23 (63.9%)
Low (400–750,000 copies/mL): n (%)	18 (46.2%)	13 (36.1%)
Suppressed (< 400 copies/mL): n (%)	0 (0%)	0 (0%)
***Other clinical / treatment measures***		
Age at ART initiation (weeks)	8.3 [7.4, 9.7] [6.6–12.0]^a^	18.9 [7.7, 27.2] [6.0–75.7]^a^
Age at ART interruption (weeks)	51.7 [47.3, 72.6]	-
Age of first viral load suppression (weeks)	33.9 [31.5, 49.8] [30.6–213.3]^a^	47.6 [33.5, 79.2] [29.1–285.6]^a^
Duration of ART interruption (weeks)	34.4 [20.4, 52.3] [5.7–299.4]^a^	0.00 (not interrupted)
Nadir CD4%	19 ± 6	20 ± 6
Age at nadir CD4% (weeks)	91.0 [55.9, 125.7]	27.9 [18.8, 51.0]
CDC classification		
A: n (%)	8 (20%)	1 (3%)
B: n (%)	9 (23%)	7 (19%)
Severe B: n (%)	2 (5%)	7 (19%)
C: n (%)	20 (51%)	19 (53%)
Unknown: n (%)	0 (0%)	2 (6%)
HIV encephalopathy diagnosis: n (%)	7 (18%)	5 (14%)

Values are mean ± standard deviation or median [IQR]

^a^range

Groups were matched for age at each time point, except at age 5 when CPHIV were 2.5 months younger on average than HIV- children. Scores on the Griffiths’ Mental Development Scales (GMDS) Extended Revision for South Africa at age 5 and on the Kaufman Assessment Battery for Children (KABC) at age 9, did not differ between CPHIV and HIV- control groups. At age 7, scores for mental processing on the KABC tended to be lower, albeit below conventional levels of significance, in CPHIV than in controls. Nonetheless, KABC developmental trajectories from age 7 to 9 were similar between CPHIV and uninfected controls (Additional file 1: Table S1). To support combining PHIV and control sub-groups in our analyses, we also present means of GMDS and KABC scores for each sub-group in Table 1. Notably, scores on the GMDS and KABC did not differ between CPHIV on Interrupted and Continuous ART, nor between CHEU and CHU at ages 5, 7 or 9 years. The number of children diagnosed with HIVE was similar in the continuous and interrupted ART groups (Table 2).

### Rate of change in CT and LGI in HIV- control children

#### Thickness

**Fig. 1:**
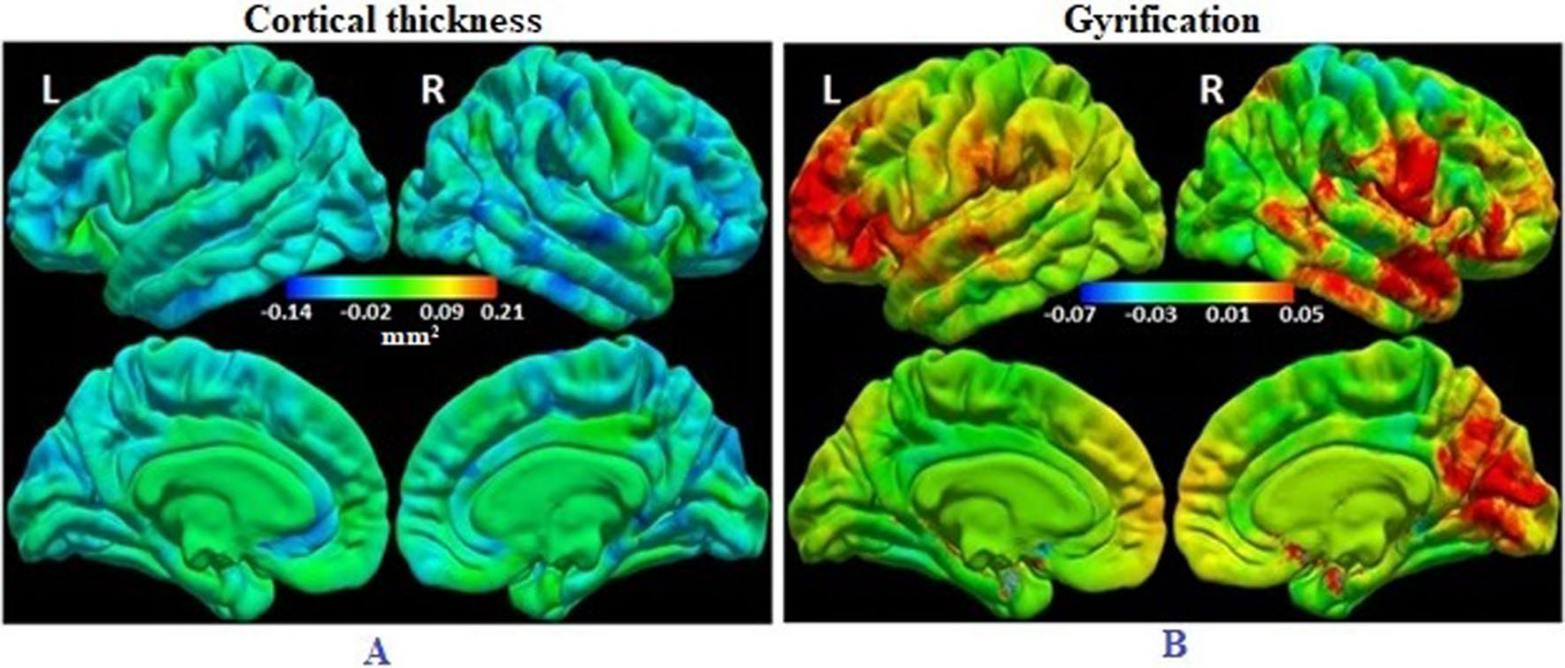
Colour map of regression coefficients showing rate of change from age 5 to 9 years of (left) cortical thickness and (right) local gyrification indices in HIV-uninfected (HIV-) children controlling for sex. Positive regression coefficients (red/yellow) indicate increases with age, while negative coefficients (cyan/blue) indicate decreases with age. The colour bar scale in each figure applies to both lateral (top) and medial (bottom) views. (N = 66 HIV- controls)

Among uninfected control children we observe widespread cortical thinning from age 5 to 9 years. This decrease in thickness was significant at an FDR-corrected *p*-value of 0.05 (β_2_ <–0.09 mm/year) over much of the lateral surface of the cortex, as well as in medial superior parietal, occipital and frontal regions. The decrease was greatest in postcentral/supramarginal, lateral frontal and temporal regions (0.11 mm/year), followed by bilateral precentral and medial occipital regions (0.07 mm/year) (Fig. 1). Adding the square of time (t_*ij*_^2^) to the model did not change the results.

#### Local gyrification indices

Children without HIV demonstrate increasing gyral folding from 5 to 9 years at an FDR-corrected *p* of 0.05 (β_2_ > 0.03) in large bilateral lateral frontal and right temporal regions, as well as right medial inferior parieto-occipital regions. The greatest changes (β_2_ in Eq. (1) = 0.047 units/year, FDR *p* < 0.001) were observed in bilateral rostral middle frontal and right superior temporal regions, and in parts of the right postcentral gyrus and parieto-occipital sulcus areas (Fig. 1). Adding the square of time (t_*ij*_^2^) to the model did not change the findings.

### Group comparison of means and rate of change of CT

**Fig. 2:**
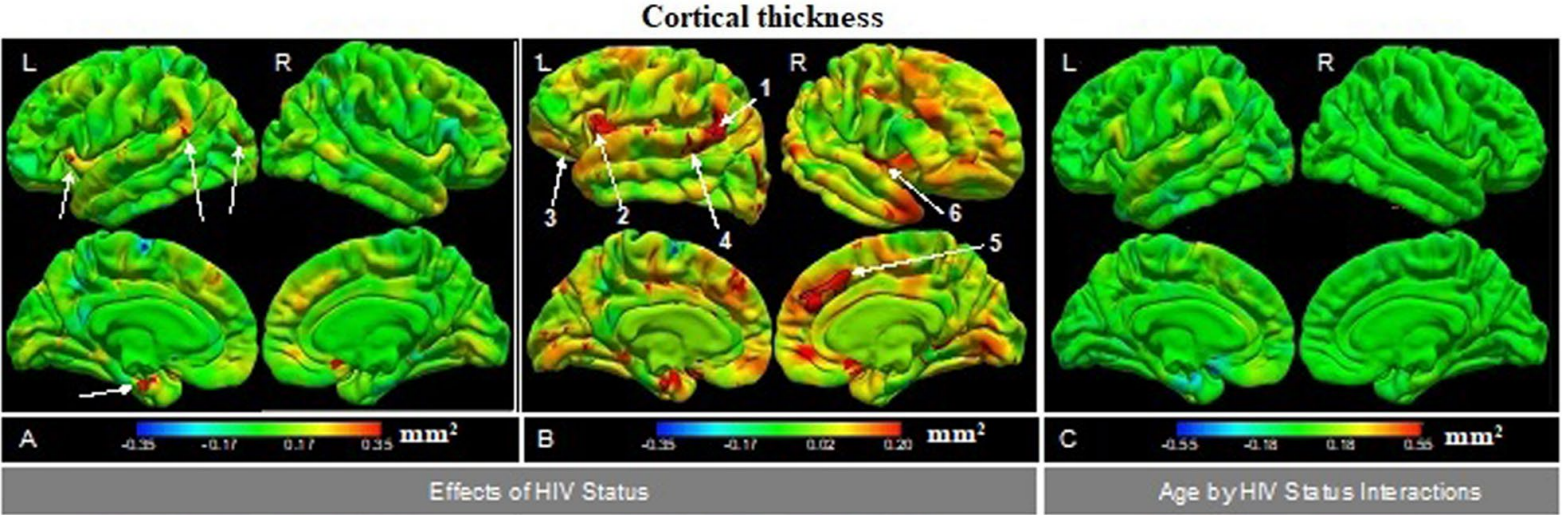
Colour map of the vertex-wise regression coefficients of the effect on cortical thickness of **A**, **B** HIV status [β_3_ in Eq. (2)] and **C** age by HIV status interactions [β_4_ in Eq. (2)], respectively. In A and B, positive regression coefficients (red/yellow) indicate thicker cortex in CPHIV compared to uninfected controls and negative coefficients (cyan/blue) thinner cortex. In **C**, positive regression coefficients (red/yellow) indicate a greater rate of change of cortical thickness from age 5 to 9 years in CPHIV than uninfected controls, and negative coefficients (cyan/blue) indicate a slower rate of change. The colour bar scale in each figure indicates the effect size (Cohen’s *d*). While the scales in **A** and **C** extend to effect sizes required for significance at FDR-corrected *p*-values, the scale in B has been right censored to highlight clusters showing group differences that survive at uncorrected *p* < 0.005. For HIV status, effect sizes ≥ 0.06 and ≥ 0.29 are significant at uncorrected and FDR corrected *p* = 0.05, respectively. For age by HIV status interactions, effect sizes ≥ 0.09 and ≥ 0.31 are significant at uncorrected and FDR corrected *p* = 0.05, respectively. (N = 141: PHIV = 75, HIV − = 66)

**Fig. 3:**
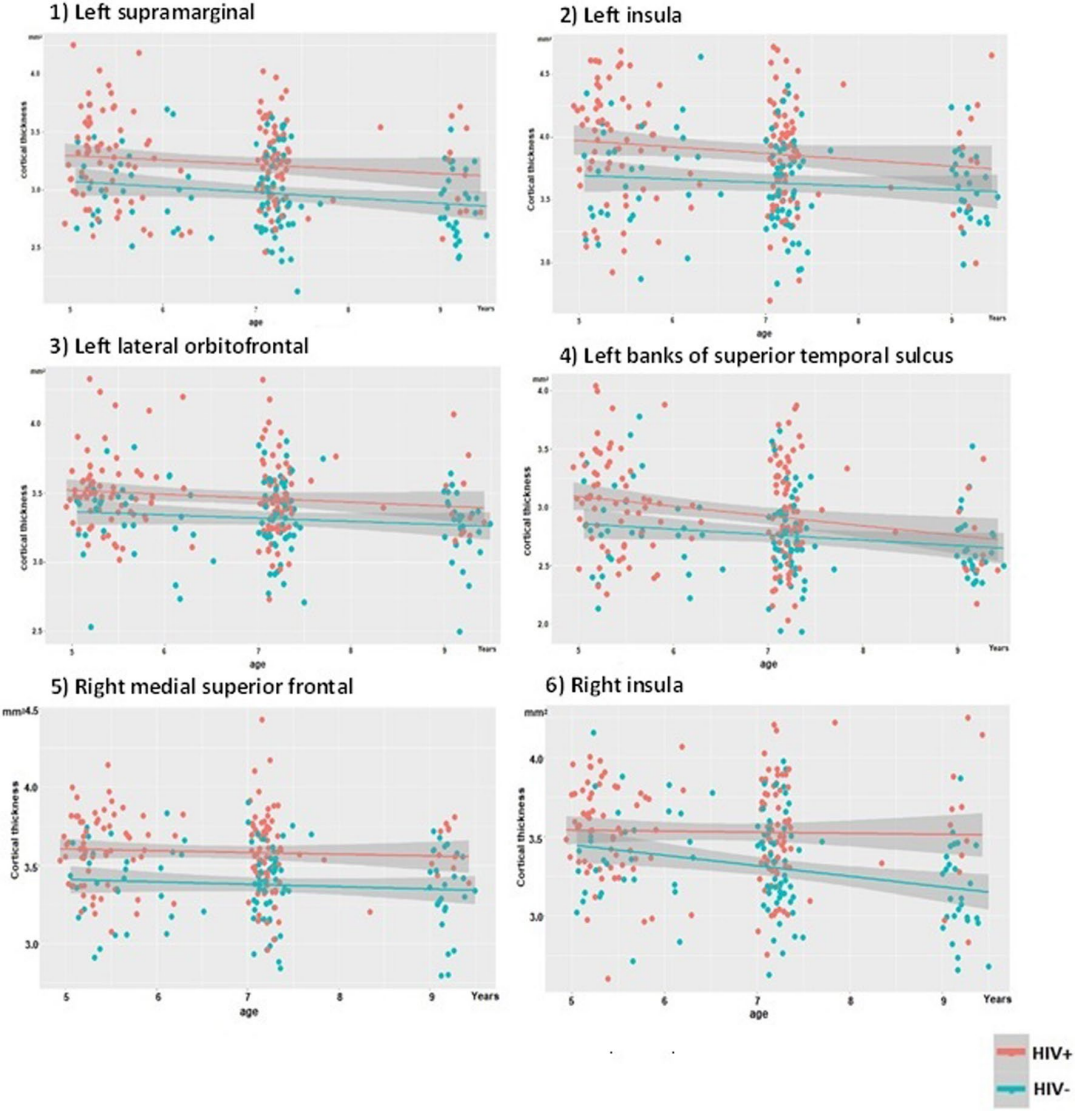
Developmental trajectories of cortical thickness from age 5 to 9 years in regions where the cortex of children with PHIV was thicker than in uninfected controls at an uncorrected *p* < 0.005. The shadows represent the 95% confidence interval (CI). (N = 141: PHIV = 75, HIV − = 66)

After correction for multiple comparisons, vertex-wise analyses did not reveal any regions where the mean CT (β_3_ in Eq. (2)), nor the rate of change of CT from age 5 to 9 years [β_4_ in Eq. (2)], differed significantly between CPHIV and their uninfected peers (Fig. 2A and C). However, small regions in the left supramarginal gyrus, insula, lateral occipital gyri, and inferior temporal gyrus (indicated by arrows in Fig. 2A) demonstrated thicker cortex in CPHIV that fell just short of significance at a strict FDR corrected threshold. To reduce the risk of type 2 errors, we identified clusters showing differences (in means or rate of change of CT) between CPHIV and HIV- controls at an uncorrected *p* < 0.005 and extracted mean CT values in these clusters for each subject at each age for which the subject provided data. At this threshold, we identified 6 regions where the cortex was thicker from age 5–9 years in CPHIV than controls (numbered arrows in Fig. 2B), but none where the rates of change of CT differed between groups. In Table 3 we present the MNI coordinates and sizes of the 6 clusters where CPHIV demonstrate thicker cortex, as well as regression coefficients from a post-hoc LME analysis. Plots of cortical thickness as a function of age are presented for each of these clusters in Fig. 3.

**Table 3:** Regions where children with PHIV have thicker cortex than HIV- controls from age 5 to 9 years at an uncorrected *p* < 0.005, and regression coefficients from a post-hoc LME analysis performed on regional means of cortical thickness extracted in these clusters for each child at each age for which the child provided data. Numbering of regression coefficients is as per Eq. (2)

Cluster region	MNI coordinates (x,y,z)	Size (mm^2^)	HIV status effects	Age effects	HIV status by age interactions	Sex effects
			β_3_ (SE) *p*-value	β_2_ (SE) *p*-value	β_4_ (SE) *p*-value	β_5_ (SE); *p*-value
L Supramarginal	− 59.0, − 46.4, 21.6	245	0.38 (0.11) < 0.001	− 0.05 (0.01) < 0.001	0.02 (0.01) 0.11	− 0.07 (0.06) 0.21
L Insula	− 32.9, 15.0, − 1.3	124	0.38 (0.16) < 0.001	− 0.06 (0.02) < 0.001	0.02 (0.02) 0.31	− 0.13 (0.07) 0.05
L Lateral orbitofrontal	− 18.0, 24.6, − 18.9	120	0.20 (0.10) < 0.001	− 0.05 (0.01) < 0.001	0.01 (0.01) 0.63	0.06 (0.04) 0.17
L Banks of Superior Temporal Sulcus	− 53.0, − 41.8, 8.6	77	0.33 (0.16) < 0.001	− 0.06 (0.01) < 0.001	0.02 (0.02) 0.46	− 0.06 (0.07) 0.34
R Medial Superior frontal	12.9, 25.3, 29.5	504	0.19 (0.08) < 0.001	− 0.02 (0.01) < 0.001	− 0.0002 (0.01) 0.98	− 0.05 (0.04) 0.19
R Insula	37.2, − 3.8, − 0.4	314	0.20 (0.12) < 0.001	− 0.03 (0.01) < 0.001	− 0.03 (0.01) 0.04	0.01 (0.05) 0.79

*L* Left; R Right; *β* unstandardised regression coefficients from LME model (Eq. 2); *SE* standard error; *p*-values are uncorrected

To ensure that our results are not biased by the absence of within-subject information for participants who provided data at only a single time point, we repeated the post-hoc LME analysis using regional CT data from *only* children who provided data at multiple time points (52 CPHIV, 33 HIV-). Although effect sizes were smaller than from analyses including all children, cortical thickening in CPHIV compared to controls remained evident in all the regions except the left banks of the superior temporal sulcus, and below conventional levels of significance in left lateral orbitofrontal cortex. Age, group by age interactions, and sex effects were similar to before (Additional file 1: Table S2). We also repeated our post-hoc LME analyses to examine whether CT developmental trajectories differed within the HIV- or PHIV sub-groups. Notably, cortical thickness development did not differ in any of the regions between exposed and unexposed uninfected control children (all *p*’s > 0.26, Additional file 1: Table S3), nor between CPHIV in whom treatment had been interrupted compared to those on continuous treatment (all *p*’s > 0.08, Additional file 1: Table S4).

We did not find any regions showing differences in the means nor rate of change of CT between either the Interrupted ART, Continuous ART, or HIVE groups and the HIV- control group at either FDR corrected *p* < 0.05 or uncorrected *p* < 0.005 thresholds. Adding the square of time (t_*ij*_^2^) to the LME model did not change the findings.

### Group comparison of means and rate of change of LGI

**Fig. 4:**
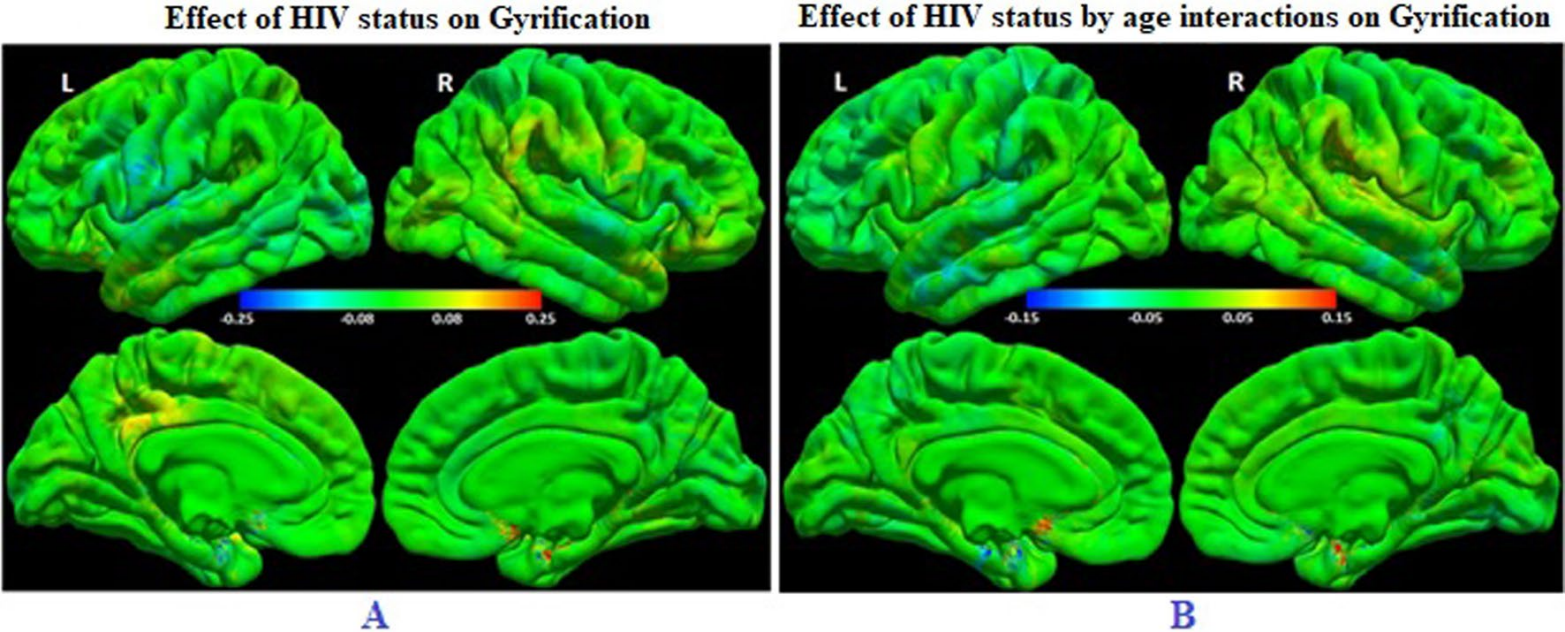
Colour map of the vertex-wise regression coefficients of the effect on local gyrification indices of **A** HIV status (β_3_ in Eq. (2)) and **B** HIV status by age interactions (β_4_ in Eq. (2)), respectively. In **A**, positive regression coefficients (red/yellow) indicate greater gyrification in CPHIV compared to uninfected controls and negative coefficients (cyan/blue) less gyrification. In **B**, positive regression coefficients (red/yellow) indicate a greater rate of change from age 5 to 9 years in CPHIV than uninfected controls, and negative coefficients (cyan/blue) indicate a slower rate of change. The colour bar scale in each figure indicates the effect size (Cohen’s *d*). For HIV status and status by age interactions, respectively, effect sizes ≥ 0.30 and ≥ 3.77 are required for significance at an FDR corrected *p* = 0.05. (N = 141: PHIV = 75, HIV- = 66)

After correction for multiple comparisons, vertex-wise analyses did not reveal any regions where the means (β_3_ in Eq. (2)), nor the rate of change from age 5 to 9 years (β_4_ in Eq. (2)), of LGI (Fig. 4A and B) differed significantly between CPHIV and their uninfected peers, nor between either the Interrupted- or Continuous ART groups and the HIV- control group. We also did not find any differences at an uncorrected *p* < 0.005. Adding the square of time (t_*ij*_^2^) to the model did not change the findings.

We did, however, find greater mean (β_3_ (SE) = 0.34 (0.12), FDR *p* = 0.04) and slower rate of change (β_4_ (SE) = − 0.03 (0.05), FDR *p* = 0.01) of gyrification in children who had previously had a diagnosis of HIVE compared to HIV- controls in a left rostral middle frontal region (MNI co-ordinates at peak: − 38.6, 45.9, 1.5; cluster size: 1702.56 mm^2^; Additional file 1: Figure S1).

## Discussion

To the best of our knowledge, this study is the first longitudinal neuroimaging investigation of cortical development in CPHIV from a resource-limited setting and who received ART before age 2 years. Similar to previous studies, typically-developing control children showed widespread decreases in cortical thickness from age 5 to 9 years [22, 42–44]. Gyrification showed less change during this period, but increases were observed in rostral middle frontal, temporal, postcentral and parieto-occipital regions. Although the developmental trajectories of cortical thickness and gyrification in CPHIV were similar to that of matched typically-developing HIV- children during this important period of brain development, we identified 6 regions, albeit at a lower significance level, where the cortex of CPHIV was thicker than in uninfected children, namely bilateral insula, left supramarginal, lateral orbitofrontal and superior temporal, and right medial superior frontal regions.

## Morphometric development in typically-developing HIV- children from age 5–9 years

Cortical thinning from childhood into early adolescence is associated with physiological processes related to neurodevelopment and regional specialization of the developing brain, and faster thinning of cortex has been associated with higher intelligence quotients (IQ) around age 10 years [45]. These physiological processes include myelinogenesis [22, 42, 46], synaptic pruning and dendritic arborisation [26, 42, 46, 47], which lead to reduction in the size and number of neurons and their synaptic processes [26, 42, 46]. These neurodevelopmental processes work together to reinforce and strengthen fibres and connections that are used consistently for transmitting brain impulses, while redundant fibres are “pruned” or removed [48]. The process of myelination in the lower cortical layer closest to the cortical white/grey matter interface may be the main process that influences cortical thinning with brain growth [22, 42, 49].

We also observed increased gyrification with age in bilateral frontal regions, which is consistent with findings by Schnack et al. [45] of increasing cortical surface area with age. Although, the developmental trajectory of cortical folding is not yet fully understood, the frontal cortex controls the complex executive skills of planning, working memory and cognitive flexibility that develops in late childhood [22, 50, 51], corresponding to the period studied here. Maturation of cortical morphology follows a back-to-front—occipital-to-prefrontal—[48] developmental sequence, with prefrontal cortex maturing last in the continuous process of development from childhood into adolescence. The regional variation in rate of change and development in brain morphometry may be related to the complexity associated with the cytoarchitecture, information processing and co-ordination functionalities of different cortical regions [22]. In terms of functional complexity, primary sensory areas change and develop faster than the more complex polymodal, higher-order association areas [22, 48, 52].

The increases in cortical folding we observed in this study may be functionally related to the acquiring of critical developmental skills in late childhood into adolescence [53, 54]. Our finding of increasing gyrification in rostral middle frontal regions through age 9 years is similar to that of Cao et al. [28], who reported that although regional gyrification decreases from medial to lateral regions starting as early as age 4, frontal gyral complexity continues to increase, possibly into the teenage years [28, 55].

## Effect of perinatal HIV infection and early ART initiation on longitudinal morphometry development

The main focus of this study was to examine whether PHIV in the context of early ART initiation affects brain development across childhood. Previous cross-sectional neuroimaging studies comparing brain developmental indices of children and youth with PHIV to healthy controls found associations of HIV infection with both regionally thicker or thinner cortex [56–59], as well as regionally-specific lower gyrification [56, 59].

In our own cross-sectional analyses of the cohort studied here, we previously reported thicker cortex at age 5 years in CPHIV compared to uninfected controls in bilateral frontal and left temporo-insular regions, and lower LGIs in left superior medial frontal and bilateral medial orbitofrontal cortices extending into rostral anterior cingulate [11]. In contrast, at age 7 years, we found thicker cortex only in a small left lateral occipital region, and less gyrification in bilateral paracentral and right temporal regions [10]. These regions seen in our cohort at ages 5 and 7 years are similar to the regions implicated in previous studies of CPHIV [56–59].

Few longitudinal studies exist of effects of HIV infection on neurodevelopment, especially in children initiated to ART early (< 2 years) who have been virally suppressed from a young age. We identified 3 longitudinal studies [57, 60, 61] that investigated brain development in children and adolescents living with HIV. Although all 3 demonstrated regionally altered brain morphometry in CPHIV, only one showed an effect of HIV on the trajectory of morphometric development [57]. Van den Hof et al. [60] reported a slower rate of normal grey matter volume decline over a period of approximately 5 years in children living with HIV aged 8—18 years, but this was attributable to variation in participant height. Although white matter volume was lower in CPHIV the developmental trajectory was no different from controls and these changes were not associated with cognitive development. Wade et al. [61] identified a subcortical pallidal region in the basal ganglia that was thinner in CPHIV with a mean age of 11 years. However, they found no effect of HIV on longitudinal shape or volume changes of the pallidum over a period of one year. By contrast, in addition to finding areas of lower GM volume and altered CT in CPHIV, Yu et al. [57] showed HIV status by age interaction effects in 13-year-old children after 1 year on GM volume, especially in bilateral parietal lobes, and on CT in the bilateral central sulcus, insula, frontal lobe and cingulate sulcus.

Overall, our findings of thicker cortex in CPHIV in bilateral insula, right medial frontal, and left supramarginal, lateral orbitofrontal and superior temporal regions (at a lower uncorrected level of significance), but similar developmental trajectories of cortical thickness and gyrification between CPHIV and HIV- children are consistent with previous studies suggesting that HIV effects on brain morphology are subtle. The slightly relaxed level of significance was to address concerns regarding insufficient power and to reduce the risk of type 2 error. Unexpectedly, only the insular clusters in this study overlap with regions where cortex was thicker at either ages 5 or 7 years in our previous studies.

The fact that brain regions implicated at either ages 7 or 9 years in our cohort do not overlap, together with the absence of HIV-related differences (at conventional levels of significance) in cortical development from 5–9 years in our current analyses, suggest that early ART initiation combined with ongoing treatment may prevent persistent HIV-associated damage to the cortex in young children. It is possible, therefore, that previously reported HIV-related alterations in CT and LGI at 5 and 7 years represent transient localized maturation differences that may be a side effect of long-term HIV infection and ongoing treatment. However, to confirm that the absence of HIV-related differences is not merely due to a lack of power, cortical development should be studied through adolescence, which will also increase power to detect long-term HIV-related differences. Notably, evidence of persistent cognitive deficits remains. At age 5 years, Beery visual perception scores were lower in CPHIV [6], and at both ages 7 and 9 years CPHIV performed worse than uninfected controls on tasks assessing executive function and auditory working memory [36].

Previously, in the same cohort studied here, we reported elevated levels of choline (glycerophosphocholine + phosphocholine) from 5 to 11 years in CPHIV compared to uninfected controls in all three regions where MR spectroscopy data were acquired, namely MFGM, BG and PWM, as well as higher myo-inositol in the MFGM [24]. Notably, the right medial superior frontal region where we find thicker cortex from age 5–9 years in CPHIV overlaps the MFGM region-of-interest (ROI) from our MRS study, and the supramarginal gyrus is adjacent to the PWM—although we find thicker cortex in the left supramarginal gyrus while the PWM ROI where MRS data were acquired was on the right. The elevated choline and myo-inositol levels in these regions suggest that the thicker cortex observed here in CPHIV may be related to persistent neuroinflammation and astrocytosis, despite early ART.

## Effect of ART interruption on morphometry development

Since children with PHIV start a lifetime of ART at birth, there is much interest in the possibility of safe treatment interruption(s). One of the goals of this follow up study was to assess the long-term implications of planned interruption compared to continuous ART. Cross-sectional comparison at age 5 years showed that children in whom ART had been interrupted had thicker cortex than HIV- controls in the left rostral and superior frontal, and right insula regions, and lower gyrification in the left precuneus and right rostral and caudal anterior cingulate regions, but higher gyrification in the right lateral occipital regions [11]. Children on continuous ART demonstrated lower gyrification compared to HIV- controls in right posterior superior frontal and superior parietal regions, but higher gyrification in left fusiform, and right rostral middle frontal and lateral occipital regions [11]. However, longitudinal analysis did not reveal any regions where CT nor LGI, nor their developmental trajectories from 5 to 9 years, differed between either CPHIV on interrupted or continuous treatment and HIV- controls.

According to previous studies, immune compromise related to a planned short ART interruption has a similar recovery pattern as continuous ART [21, 62]. Research has shown infants’ immune systems to be dynamic and malleable, allowing them to recover from short periods of ART interruption [19, 21]. Several studies demonstrate that the most important factors in perinatal HIV treatment is ART initiation timing and duration on treatment before interruption [20, 34, 62]. Early ART initiation is beneficial for improved immune system and viral suppression, as well as improving neurodevelopmental outcomes [2, 8, 34]. These results suggest that when ART is initiated before critical developmental processes are affected by viral replication, a short interruption may not affect long-term immune health. In contrast, a longer interruption may lead to viral rebound, resistance and neuropsychological consequences [19, 20, 63]. The range of interruption durations for the children in the cohort under study is quite large (6–300 weeks). Although ART interruption may lead to age specific alterations in brain morphometry development [11], the absence of HIV-related differences in the means and developmental trajectories of CT and LGI suggest that differences do not persist to later ages.

## Effect of HIVE on morphometry development

With the introduction of early ART, there has been a decline in the incidence of HIVE [64, 65]. In our cohort of early-treated CPHIV, only 16% had been diagnosed with HIV-related encephalopathy, compared to incidences of 20–60% in previous studies of perinatally infected but ART-naïve pediatric cohorts [64, 66, 67]. We did not find any differences in the developmental trajectories of cortical thickness or gyrification between the HIVE group and HIV- controls, except in the left rostral middle frontal region where children with HIVE demonstrated greater mean gyrification than HIV- controls and failed to show the age-related increase in gyrification seen in controls. Although the small number of HIVE subjects raises concerns regarding the reliability of this finding, the low number of HIVE children in our cohort and the limited number of regions found to show altered developmental trajectories, reinforce the benefits of early ART in reducing the occurrence and severity of neurologic conditions such as HIVE.

Since brain development moves from posterior (occipital lobe) to anterior (frontal lobe), the frontal lobe of the brain is expected to develop last [49]. The reduced age-related changes in gyrification in the rostral middle frontal region in HIVE children may therefore point to developmental delay. However, it may simply be a consequence of the higher gyrification evident in HIVE children in this region at age 5 years.

## Limitations and future work

The small sample size at age 9 years, with fewer CPHIV compared to HIV- controls, was due to the decommissioning of the 3 T Siemens *Allegra* brain scanner used for this study. The small number of children diagnosed with HIVE precludes strong conclusions about group differences. Although FreeSurfer’s longitudinal pipeline was not originally designed for pediatric cortical segmentation, manual quality checks were performed on the cortical model to ensure accurate extraction and segmentation of cortical surfaces.

A key theory of cortical folding formation links gyral and sulcal formation in the brain to neural connectivity [52]. It postulates that regions with greater neural connectivity are associated with greater tension, ensuring that such brain regions are in close proximity during brain growth, forming gyri, while regions with lower connectivity follow a similar pattern to form sulci [47, 52]. Functional connections are associated with acquiring new skills and abilities during neurodevelopment, leading to changes in surface morphology of the cortex. Hence it may be worthwhile for future longitudinal studies to investigate gyrification development in relation to neural connections using DTI and functional connectivity measures.

The relationship between cortical developmental trajectories and longitudinal neuropsychological outcomes should also be examined.

## Conclusion

We present results from a follow-up neuroimaging study of a subset of children from the CHER trial during an important stage of neurodevelopment (5–9 years). In our control group, generalized cortical thinning was observed from age 5 to 9 years, with the rate of thinning varying by region. Age-related increases in gyrification were observed in large bilateral frontal, right temporal, and right medial inferior parieto-occipital regions. After correction for multiple comparisons, we did not find any regions where the means or developmental trajectories of cortical thickness and gyrification differed between CPHIV who initiated ART early and uninfected controls. However, at an uncorrected *p* < 0.005, we found 6 regions where CPHIV had thicker cortex than uninfected controls. Planned ART interruption did not affect development of cortical morphometry. HIV-related encephalopathy was associated with greater gyrification and the absence of age-related gyrification increases in bilateral rostral middle frontal regions. Although our results suggest that early ART initiation preserves normal development of cortical morphometry between the ages of 5 and 9 years in perinatal HIV infection, these findings need to be confirmed with longitudinal follow-up through the vulnerable adolescent period.

## Supplementary Information

**Additional file 1: Table S1**. Regression coefficients from a linear mixed effect analysis examining effects of HIV status on KABC developmental trajectories from age 7 to 9 years. **Table S2.** Regression coefficients from a post-hoc LME analysis of CT trajectories in regions where CPHIV showed cortical thickening using *only* children who provided data at more than one time point (N=85; 52 PHIV). Numbering of regression coefficients is as per equation (2). **Table S3.** Regression coefficients from a post-hoc LME analysis comparing CT developmental trajectories between CHEU and CHU in the 6 regions where children with PHIV demonstrated thicker cortex from age 5 to 9 years than HIV- controls. Numbering of regression coefficients is as per equation (2). **Table S4.** Regression coefficients from a post-hoc LME analysis comparing CT developmental trajectories between CPHIV in whom treatment was interrupted and those on continuous ART in the 6 regions where children with PHIV demonstrated thicker cortex from age 5 to 9 years than HIV- controls. Numbering of regression coefficients is as per equation (2). **Figure S1**. Effects of HIV status (HIVE vs HIV-) by age interactions on local gyrification indices (LGIs). (A) Colour map of effects sizes. Positive regression coefficients (red/yellow) indicate greater rates of change of LGI in children who had previously been diagnosed with HIVE than HIV-, and negative coefficients (cyan/blue) indicate lower rates of change in HIVE than HIV- children. The colour bar scale applies to both lateral (top) and medial (bottom) views. Children with a previous diagnosis of HIVE did not demonstrate the increasing gyrification from ages 5 to 9 years evident in HIV- controls in bilateral rostral middle frontal regions. In the left hemisphere in the region outlined in red (white arrow), the difference is significant at an FDR-corrected *p*<0.05. (B) Plots showing the rate of change of gyrification in the left rostral middle frontal regions from ages 5 and 9 across HIVE and HIV- groups (left) and a spaghetti plot of individual trajectories (right). (HIV- = 66, HIVE = 12).

## Data Availability

The datasets used and/or analysed during the current study are available from the corresponding authors on reasonable request.

## Abbreviations

ART: Antiretrovial therapy
BG: Basal ganglia
cART: Combination ART
CD4: Cluster of differentiation 4 (Glycoprotein in immune cells)
CHER: Children with HIV Early antiRetroviral therapy
CHEU: Children who are HIV exposed and uninfected
CHU: Children who are HIV unexposed
CNS: Central nervous system
CPHIV: Children perinatally infected with HIV
CT: Cortical thickness
DTI: Diffusion tensor imaging
EPI: Echo planar imaging
FAMCRU: FAMily Centre for Research with Ubuntu, Tygerberg Children’s Hospital Cape Town, South Africa
FDR: False discovery rate
FOV: Field of view
FWHM: Full-width, half-maximum
HIV: Human immunodeficiency virus
HIVE: HIV-related encephalopathy
LGI: Local gyrification index
LME: Linear mixed effect model
LPR/r: Lopinavir-Ritonavir
MATLAB: MATrix LABoratory (programming language)
MEMPRAGE: MultiEcho Magnetization Prepared RApid Gradient Echo
MFGM: MidFrontal gray matter
MRI: Magnetic resonance imaging
MRS: Magnetic resonance spectroscopy
PHIV: Perinatal HIV
PWM: Peritrigonal white matter
ROI: Region of interest
SD: Standard deviation
SSA: Sub-Saharan Africa
TR: Repetition time
TI: Inversion time
TE: Time-to-echo
ZDV: Zidovudine
3D: 3-dimensional
3 T: 3 Tesla MRI
3TC: Lamivudine

## References

[1] UNAIDS. Global HIV & AIDS statistics—Fact sheet. 2022. Source: 2022. https://www.unaids.org/en/resources/fact-sheet. Accessed Aug 24.

[2] Violari A, Cotton MF, Gibb DM, Babiker AG, Steyn J, Madhi SA (2008). Early antiretroviral therapy and mortality among HIV-infected infants. N Engl J Med.

[3] Lindsey JC, Malee KM, Brouwers P, Hughes MD (2007). Neurodevelopmental functioning in HIV-infected infants and young children before and after the introduction of protease inhibitor–based highly active antiretroviral therapy. Pediatrics.

[4] World Health Organization (WHO) (2008). Report of the WHO technical reference group. Paediatric HIV/ART care guideline group meeting.

[5] Van Rie A, Harrington PR, Dow A, Robertson K (2007). Neurologic and neurodevelopmental manifestations of pediatric HIV/AIDS: a global perspective. Eur J Paediatr Neurol.

[6] Laughton B, Cornell M, Kidd M, Springer PE, Dobbels EFMT, Rensburg AJV (2018). Five year neurodevelopment outcomes of perinatally HIV-infected children on early limited or deferred continuous antiretroviral therapy. J Int AIDS Soc.

[7] Laughton B, Cornell M, Boivin M, Van Rie A (2013). Neurodevelopment in perinatally HIV-infected children: a concern for adolescence. J Int AIDS Soc.

[8] Laughton B, Cornell M, Grove D, Kidd M, Springer PE, Dobbels E (2012). Early antiretroviral therapy improves neurodevelopmental outcomes in infants. AIDS (London, England).

[9] Randall SR, Warton CM, Holmes MJ, Cotton MF, Laughton B, van der Kouwe AJ (2017). Larger subcortical gray matter structures and smaller corpora callosa at age 5 years in HIV infected children on early ART. Front Neuroanat.

[10] Nwosu EC, Robertson FC, Holmes MJ, Cotton MF, Dobbels E, Little F (2018). Altered brain morphometry in 7-year old HIV-infected children on early ART. Metab Brain Dis.

[11] Nwosu EC, Holmes MJ, Cotton MF, Dobbels E, Little F, Laughton B (2021). Cortical structural changes related to early antiretroviral therapy (ART) interruption in perinatally HIV-infected children at 5 years of age. IBRO Neuroscience Reports.

[12] Mbugua KK, Holmes MJ, Cotton MF, Ratai EM, Little F, Hess AT (2016). HIV-associated CD4/8 depletion in infancy is associated with neurometabolic reductions in the basal ganglia at age 5 years despite early antiretroviral therapy. AIDS.

[13] Robertson FC, Holmes MJ, Cotton MF, Dobbels E, Little F, Laughton B (2018). Perinatal HIV infection or exposure is associated with low N-acetylaspartate and glutamate in Basal Ganglia at age 9 but not 7 Years. Front Hum Neurosci.

[14] Graham AS, Holmes MJ, Little F, Dobbels E, Cotton MF, Laughton B (2020). MRS suggests multi-regional inflammation and white matter axonal damage at 11 years following perinatal HIV infection. Neuro Image Clin.

[15] Ackermann C, Andronikou S, Saleh MG, Laughton B, Alhamud AA, van der Kouwe A (2016). Early antiretroviral therapy in HIV-infected children is associated with diffuse white matter structural abnormality and corpus callosum sparing. Am J Neuroradiol.

[16] Jankiewicz M, Holmes MJ, Taylor PA, Cotton MF, Laughton B, van der Kouwe AJ (2017). White matter abnormalities in children with HIV infection and exposure. Front Neuroanat.

[17] Li JZ, Smith DM, Mellors JW (2015). The critical roles of treatment interruption studies and biomarker identification in the search for an HIV cure. AIDS.

[18] Dubrocq G, Rakhmanina N (2018). Antiretroviral therapy interruptions: impact on HIV treatment and transmission. HIV/AIDS.

[19] Wamalwa D, Benki-Nugent S, Langat A, Tapia K, Ngugi E, Moraa H (2016). Treatment interruption after 2-year antiretroviral treatment initiated during acute/early HIV in infancy. AIDS.

[20] Ananworanich J, Melvin D, Amador JT, Childs T, Medin G, Boscolo V (2016). Neurocognition and quality of life after reinitiating antiretroviral therapy in children randomized to planned treatment interruption. AIDS.

[21] Lewis J, Payne H, Walker AS, Otwombe K, Gibb DM, Babiker AG (2017). Thymic output and CD4 T-cell reconstitution in HIV-infected children on early and interrupted antiretroviral treatment: evidence from the children with HIV early antiretroviral therapy trial. Front Immunol.

[22] Shaw P, Kabani NJ, Lerch JP, Eckstrand K, Lenroot R, Gogtay N (2008). Neurodevelopmental trajectories of the human cerebral cortex. J Neurosci.

[23] Reuter M, Schmansky NJ, Rosas HD, Fischl B (2012). Within-subject template estimation for unbiased longitudinal image analysis. Neuroimage.

[24] Van Biljon N, Robertson F, Holmes M, Cotton MF, Laughton B, van der Kouwe A (2021). Multivariate approach for longitudinal analysis of brain metabolite levels from ages 5–11 years in children with perinatal HIV infection. Neuroimage.

[25] Moeskops P, Benders MJ, Kersbergen KJ, Groenendaal F, de Vries LS, Viergever MA (2015). Development of cortical morphology evaluated with longitudinal MR brain images of preterm infants. PLoS ONE.

[26] Mills KL, Tamnes CK (2014). Methods and considerations for longitudinal structural brain imaging analysis across development. Dev Cogn Neurosci.

[27] Fischl B, Dale AM (2000). Measuring the thickness of the human cerebral cortex from magnetic resonance images. Proc Natl Acad Sci.

[28] Cao B, Mwangi B, Passos IC, Wu MJ, Keser Z, Zunta-Soares GB (2017). Lifespan gyrification trajectories of human brain in healthy individuals and patients with major psychiatric disorders. Sci Rep.

[29] Li G, Wang L, Shi F, Lyall AE, Lin W, Gilmore JH (2014). Mapping longitudinal development of local cortical gyrification in infants from birth to 2 years of age. J Neurosci.

[30] Lebel C, Beaulieu C (2011). Longitudinal development of human brain wiring continues from childhood into adulthood. J Neurosci.

[31] Aubert-Broche B, Fonov VS, García-Lorenzo D, Mouiha A, Guizard N, Coupé P (2013). A new method for structural volume analysis of longitudinal brain MRI data and its application in studying the growth trajectories of anatomical brain structures in childhood. Neuroimage.

[32] Tamnes CK, Walhovd KB, Dale AM, Østby Y, Grydeland H, Richardson G (2013). Brain development and aging: overlapping and unique patterns of change. Neuroimage.

[33] World Health Organization (WHO). Antiretroviral therapy of HIV infection in infants and children in resource-limited settings: towards universal access. Recommendations for a public health approach. 2006. Source:http://www.who.int/hiv/pub/guidelines/WHOpaediatric.pdf. Accessed 12 October, 2017.

[34] Cotton MF, Violari A, Otwombe K, Panchia R, Dobbels E, Rabie H (2013). Early time-limited antiretroviral therapy versus deferred therapy in South African infants infected with HIV: results from the children with HIV early antiretroviral (CHER) randomised trial. Lancet.

[35] Madhi SA, Adrian P, Cotton MF, McIntyre JA, Jean-Philippe P, Meadows S (2010). Effect of HIV infection status and anti-retroviral treatment on quantitative and qualitative antibody responses to pneumococcal conjugate vaccine in infants. J Infect Dis.

[36] van Wyhe KS, Laughton B, Cotton MF, Meintjes EM, van der Kouwe AJ, Boivin MJ (2021). Cognitive outcomes at ages seven and nine years in South African children from the children with HIV early antiretroviral (CHER) trial: a longitudinal investigation. J Int AIDS Soc.

[37] Tisdall MD, Hess AT, Reuter M, Meintjes EM, Fischl B, van der Kouwe AJ (2012). Volumetric navigators for prospective motion correction and selective reacquisition in neuroanatomical MRI. Magn Reson Med.

[38] Van der Kouwe AJ, Benner T, Salat DH, Fischl B (2008). Brain morphometry with multiecho MPRAGE. Neuroimage.

[39] Reuter M, Rosas HD, Fischl B (2010). Highly accurate inverse consistent registration: a robust approach. Neuroimage.

[40] Reuter M, Fischl B (2011). Avoiding asymmetry-induced bias in longitudinal image processing. Neuroimage.

[41] Bernal-Rusiel JL, Greve DN, Reuter M, Fischl B, Sabuncu MR (2013). Alzheimer’s disease neuroimaging initiative. Statistical analysis of longitudinal neuroimage data with linear mixed effects models. Neuroimage.

[42] Sowell ER, Thompson PM, Leonard CM, Welcome SE, Kan E, Toga AW (2004). Longitudinal mapping of cortical thickness and brain growth in normal children. J Neurosci.

[43] Raznahan A, Shaw P, Lalonde F, Stockman M, Wallace GL, Greenstein D (2011). How does your cortex grow?. J Neurosci.

[44] Ducharme S, Albaugh MD, Nguyen TV, Hudziak JJ, Mateos-Pérez JM, Labbe A (2016). Trajectories of cortical thickness maturation in normal brain development—the importance of quality control procedures. Neuroimage.

[45] Schnack HG, Van Haren NE, Brouwer RM, Evans A, Durston S, Boomsma DI (2015). Changes in thickness and surface area of the human cortex and their relationship with intelligence. Cereb Cortex.

[46] Sowell ER, Peterson BS, Thompson PM, Welcome SE, Henkenius AL, Toga AW (2003). Mapping cortical change across the human life span. Nat Neurosci.

[47] White T, Su S, Schmidt M, Kao CY, Sapiro G (2010). The development of gyrification in childhood and adolescence. Brain Cogn.

[48] Santos E, Noggle CA, Goldstein S, Naglieri JA (2011). Synaptic pruning. Encyclopedia of child behavior and development.

[49] Gogtay N, Giedd JN, Lusk L, Hayashi KM, Greenstein D, Vaituzis AC (2004). Dynamic mapping of human cortical development during childhood through early adulthood. Proc Natl Acad Sci.

[50] Huizinga M, Dolan CV, van der Molen MW (2006). Age-related change in executive function: developmental trends and a latent variable analysis. Neuropsychologia.

[51] Diamond A, Stuss D, Knight R (2002). Normal development of prefrontal cortex from birth to young adulthood: cognitive functions, anatomy, and biochemistry. Principles of frontal lobe function.

[52] Van Essen DC (1997). A tension-based theory of morphogenesis and compact wiring in the central nervous system. Nature.

[53] Chung YS, Hyatt CJ, Stevens MC (2017). Adolescent maturation of the relationship between cortical gyrification and cognitive ability. Neuroimage.

[54] Kersbergen KJ, Leroy F, Išgum I, Groenendaal F, de Vries LS, Claessens NH (2016). Relation between clinical risk factors, early cortical changes, and neurodevelopmental outcome in preterm infants. Neuroimage.

[55] Blanton RE, Levitt JG, Thompson PM, Narr KL, Capetillo-Cunliffe L, Nobel A (2001). Mapping cortical asymmetry and complexity patterns in normal children. Psychiatr Res Neuroimaging.

[56] Lewis-de Los Angeles CP, Williams PL, Jenkins LM, Huo Y, Malee K, Alpert KI (2020). Brain morphometric differences in youth with and without perinatally-acquired HIV: a cross-sectional study. NeuroImage Clinical..

[57] Yu X, Gao L (2019). Neuroanatomical changes underlying vertical HIV infection in adolescents. Front Immunol.

[58] Yadav SK, Gupta RK, Garg RK, Venkatesh V, Gupta PK, Singh AK (2017). Altered structural brain changes and neurocognitive performance in pediatric HIV. Neuro Image Clin.

[59] Hoare J, Fouche JP, Phillips N, Joska JA, Myer L, Zar HJ (2018). Structural brain changes in perinatally HIV-infected young adolescents in South Africa. AIDS.

[60] Van den Hof M, Jellema PE, Ter Haar AM, Scherpbier HJ, Schrantee A, Kaiser A, Caan MW, Majoie CB, Reiss P, Wit FW, Mutsaerts HJ (2021). Normal structural brain development in adolescents treated for perinatally acquired HIV: a longitudinal imaging study. AIDS.

[61] Wade BS, Valcour VG, Puthanakit T, Saremi A, Gutman BA, Nir TM, Watson C, Aurpibul L, Kosalaraksa P, Ounchanum P, Kerr S (2019). Mapping abnormal subcortical neurodevelopment in a cohort of Thai children with HIV. NeuroImage Clin..

[62] Bunupuradah T, Duong T, Compagnucci A, McMaster P, Bernardi S, Kanjanavanit S (2013). Outcomes after reinitiating antiretroviral therapy in children randomized to planned treatment interruptions. AIDS.

[63] Montserrat M, Plana M, Guardo AC, Andrés C, Climent N, Gallart T (2017). Impact of long-term antiretroviral therapy interruption and resumption on viral reservoir in HIV-1 infected patients. AIDS.

[64] Donald KA, Hoare J, Eley B, Wilmshurst JM (2014). Neurologic complications of pediatric human immunodeficiency virus: implications for clinical practice and management challenges in the African setting. Semin Pediatr Neurol.

[65] Chriboga CA, Fleishman S, Champion S, Gaye-Robinson L, Abrams EJ (2005). Incidence and prevalence of HIV encephalopathy in children with HIV infection receiving highly active anti-retroviral therapy (HAART). J Pediatr.

[66] Foster CJ, Biggs RL, Melvin D, Walters MDS, Tudor-Williams G, Lyall EGH (2006). Neurodevelopmental outcomes in children with HIV infection under 3 years of age. Dev Med Child Neurol.

[67] Lobato MN, Caldwell MB, Ng P, Oxtoby MJ (1995). Pediatric spectrum of disease clinical consortium. Encephalopathy in children with perinatally acquired human immunodeficiency virus infection. J Pediatr..

