# Correction to: Similar cortical morphometry trajectories from 5 to 9 years in children with perinatal HIV who started treatment before age 2 years and uninfected controls

**DOI:** 10.1186/s12868-023-00802-7

**Published:** 2023-07-12

**Authors:** Emmanuel C. Nwosu, Martha J. Holmes, Mark F. Cotton, Els Dobbels, Francesca Little, Barbara Laughton, Andre van der Kouwe, Frances Robertson, Ernesta M. Meintjes

**Affiliations:** 1 Division of Biomedical Engineering, Department of Human Biology, Faculty of Health Sciences, Biomedical Engineering Research Centre, University of Cape Town, Anzio Road, Observatory, Cape Town, 7925 South Africa; 2 Neuroscience Institute, University of Cape Town, Cape Town, South Africa; 3 Department of Pediatrics and Child Health, Family Centre for Research With Ubuntu (FAMCRU), Tygerberg Hospital, Stellenbosch University, Cape Town, South Africa; 4 Department of Statistical Sciences, University of Cape Town, Cape Town, South Africa; 5 A. A. Martinos Centre for Biomedical Imaging, Massachusetts General Hospital, Boston, MA USA; 6 Department of Radiology, Harvard Medical School, Boston, MA USA; 7 Cape Universities Body Imaging Centre, University of Cape Town, Cape Town, South Africa

**Correction to: BMC Neuroscience (2023) 24:15** **https://doi.org/10.1186/s12868-023-00783-7**

Following publication of the original article [1], the authors identified errors in Table 1 and 2. The correct Tables are given hereafter.

**Table 1:** Biographical data for all participants (N = 141; 75 PHIV, 66 HIV-)

	CPHIV			HIV-			*t*/χ^2^		*p*
**Age: 5 years (N = 82)**									
Sample size (N)	52			30					
Age at scan (years)	5.39 ± 0.31			5.60 ± 0.43			− 2.64		**0.01**
Number of males (% males)	21 (40%)			16 (53%)			1.37		0.24
Birth weight (g)	3125 ± 386			2973 ± 603			1.47		0.14
Estimated total intracranial volume (ETIV) (cm^3^)	1375 ± 96			1363 ± 110			0.53		0.60
Neuropsychological measures GMDS^a^	All CPHIV	IntART (n = 24)	ConART (n = 28)	All HIV-	CHEU (n = 17)	CHU (n = 13)			
Performance (EQ)	74 ± 10	73 ± 11	76 ± 10	76 ± 18	73 ± 20	79 ± 17	− 0.55		0.58
Practical reasoning (FQ)	77 ± 8	76 ± 10	77 ± 7	76 ± 11	73 ± 12	79 ± 7	0.60		0.55
Sub-scales aggregate (GQ)	83 ± 6	83 ± 7	83 ± 6	83 ± 8	82 ± 10	84 ± 6	0.23		0.82
**Age: 7 years (N = 125)**									
Sample size (N)	71			54					
Age at scan (years)	7.20 ± 0.13			7.24 ± 0.13			− 1.40		0.16
Number of males (% males)	36 (51%)			30 (56%)			0.13		0.72
Birth weight (g)	3081 ± 454			3081 ± 481			− 0.01		0.99
Estimated total intracranial volume (ETIV) (cm^3^)	1419 ± 101			1444 ± 137			− 1.17		0.24
Neuropsychological measures KABC^b^ COMPOSITE SCORES	All CPHIV	IntART (n = 32)	ConART (n = 39)	All HIV-	CHEU (n = 23)	CHU (n = 31)			
Mental processing index	72 ± 8	72 ± 8	73 ± 8	75 ± 10	74 ± 10	76 ± 11	− 1.90		0.06
Non-verbal index	74 ± 11	73 ± 11	75 ± 11	76 ± 12	73 ± 12	78 ± 11	− 0.96		0.34
**Age: 9 years (N = 41)**									
Sample size (N)	12			29					
Age at scan (years)	9.13 ± 0.27			9.03 ± 0.56			0.58		0.57
Number of males (% males)	7 (58%)			16 (55%)			< 0.001		1.00
Birth weight (g)	3126 ± 479			3171 ± 466			− 0.28		0.78
Estimated total intracranial volume (ETIV) (cm^3^)	1422 ± 683			1437 ± 135			− 0.38		0.71
Neuropsychological measures KABC^b^ COMPOSITE SCORES	All CPHIV	IntART (n = 8)	ConART (n = 4)	All HIV-	CHEU (n = 17)	CHU (n = 12)			
Mental processing index	73 ± 10	73 ± 10	72 ± 10	79 ± 12	77 ± 12	81 ± 12	− 1.49		0.14
Non-verbal index	78 ± 10	78 ± 8	77 ± 15	80 ± 14	80 ± 14	80 ± 13	− 0.46		0.65

All values are mean ± standard deviation except where indicated otherwise; Bold indicates significance at *p* < 0.05

*CPHIV* children with perinatal HIV; HIV- children without HIV, *IntART* children with PHIV in whom ART was interrupted; *ConART* children with PHIV who received continuous ART; *CHEU* uninfected children who were exposed to HIV; *CHU* uninfected children unexposed to HIV

^a^Griffiths’ Mental Development Scales—extended revised: 2–8 years

^b^Kaufman Assessment Battery for Children, second edition

**Table 2:** Clinical data for all children with PHIV (N = 75)

	**Interrupted ART**	**Continuous ART**
Sample size (N)	39	36
***Clinical data at baseline (age 6–8 weeks)***		
CD4 count (cells/mm^3^)	1827 ± 955	1739 ± 833
CD4%	33 ± 10	33 ± 10
CD4/CD8 ratio	1.4 ± 0.9 [0.2–4.4]^a^	1.2 ± 0.7 [0.2–3.4]^a^
CD8 count (cells/mm^3^)	1660 ± 1218	1686 ± 871
Viral load at enrolment		
High (>750,000 copies/mL): n (%)	21 (53.8%)	23 (63.9%)
Low (400–750,000 copies/mL): n (%)	18 (46.2%)	13 (36.1%)
Suppressed (< 400 copies/mL): n (%)	0 (0%)	0 (0%)
***Other clinical / treatment measures***		
Age at ART initiation (weeks)	8.3 [7.4, 9.7] [6.6–12.0]^a^	18.9 [7.7, 27.2] [6.0–75.7]^a^
Age at ART interruption (weeks)	51.7 [47.3, 72.6]	-
Age of first viral load suppression (weeks)	33.9 [31.5, 49.8] [30.6–213.3]^a^	47.6 [33.5, 79.2] [29.1–285.6]^a^
Duration of ART interruption (weeks)	34.4 [20.4, 52.3] [5.7–299.4]^a^	0.00 (not interrupted)
Nadir CD4%	19 ± 6	20 ± 6
Age at nadir CD4% (weeks)	91.0 [55.9, 125.7]	27.9 [18.8, 51.0]
CDC classification		
A: n (%)	8 (20%)	1 (3%)
B: n (%)	9 (23%)	7 (19%)
Severe B: n (%)	2 (5%)	7 (19%)
C: n (%)	20 (51%)	19 (53%)
Unknown: n (%)	0 (0%)	2 (6%)
HIV encephalopathy diagnosis: n (%)	7 (18%)	5 (14%)

Values are mean ± standard deviation or median [IQR]

^a^range

The original article [1] has been updated.
