# Supplementary material for: Similar cortical morphometry trajectories from 5 to 9 years in children with perinatal HIV who started treatment before age 2 years and uninfected controls
Source: BMC Neurosci. 2023 Feb 24;24:15. doi: 10.1186/s12868-023-00783-7 (PMC9951512; doi:10.1186/s12868-023-00783-7)
Supplement: Supplementary file 1 — Additional file 1: Table S1. Regression coefficients from a linear mixed effect analysis examining effects of HIV status on KABC developmental trajectories from age 7 to 9 years. Table S2. Regression coefficients from a post-hoc LME analysis of CT trajectories in regions where CPHIV showed cortical thickening using only children who provided data at more than one time point (N=85; 52 PHIV). Numbering of regression coefficients is as per equation (2). Table S3. Regression coefficients from a post-hoc LME analysis comparing CT developmental trajectories between CHEU and CHU in the 6 regions where children with PHIV demonstrated thicker cortex from age 5 to 9 years than HIV- controls. Numbering of regression coefficients is as per equation (2). Table S4. Regression coefficients from a post-hoc LME analysis comparing CT developmental trajectories between CPHIV in whom treatment was interrupted and those on continuous ART in the 6 regions where children with PHIV demonstrated thicker cortex from age 5 to 9 years than HIV- controls. Numbering of regression coefficients is as per equation (2). Figure S1. Effects of HIV status (HIVE vs HIV-) by age interactions on local gyrification indices (LGIs). (A) Colour map of effects sizes. Positive regression coefficients (red/yellow) indicate greater rates of change of LGI in children who had previously been diagnosed with HIVE than HIV-, and negative coefficients (cyan/blue) indicate lower rates of change in HIVE than HIV- children. The colour bar scale applies to both lateral (top) and medial (bottom) views. Children with a previous diagnosis of HIVE did not demonstrate the increasing gyrification from ages 5 to 9 years evident in HIV- controls in bilateral rostral middle frontal regions. In the left hemisphere in the region outlined in red (white arrow), the difference is significant at an FDR-corrected p<0.05. (B) Plots showing the rate of change of gyrification in the left rostral middle frontal regions from ages 5 and 9 acros [file 12868_2023_783_MOESM1_ESM.docx]

**Additional files**

**Table S1**: Regression coefficients from a linear mixed effect analysis examining effects of HIV status on KABC developmental trajectories from age 7 to 9 years.

| Cluster region | HIV status effects | Age effects | HIV status by age interactions | Sex effects |
| --- | --- | --- | --- | --- |
|  |  |  |  |  |
|  | β_3_  (SE)  *p*-value | β_2_  (SE)  *p*-value | β_4_  (SE)  *p*-value | β_5_  (SE)  *p*-value |
| Mental Processing Index | 12.97 (12.47)  0.11 | 2.68 (1.36)  0.03 | -1.35  (1.64)  0.41 | -2.03  (1.70)  0.23 |
| Non-verbal Index | -7.74 (11.84)  0.32 | -0.90 (1.32)  0.96 | 1.29  (1.56)  0.41 | -3.21  (1.92)  0.10 |

**Table S2:** Regression coefficients from a post-hoc LME analysis of CT trajectories in regions where CPHIV showed cortical thickening using *only* children who provided data at more than one time point (N=85; 52 PHIV). Numbering of regression coefficients is as per equation (2).

| Cluster region | HIV status effects | Age effects | HIV status by age interactions | Sex effects |
| --- | --- | --- | --- | --- |
|  |  |  |  |  |
|  | β_3_  (SE)  *p*-value | β_2_  (SE)  *p*-value | β_4_  (SE)  *p*-value | β_5_  (SE)  *p*-value |
| L Supramarginal | 0.38  (0.12)  0.003 | -0.05  (0.01)  <0.001 | 0.02  (0.01)  0.11 | -0.08  (0.08)  0.31 |
| L Insula | 0.36  (0.17)  0.02 | -0.06  (0.02)  <0.001 | 0.02  (0.02)  0.30 | -0.09  (0.08)  0.28 |
| L Lateral orbitofrontal | 0.09  (0.10)  0.09 | -0.04  (0.01)  <0.001 | 0.001  (0.01)  0.94 | 0.08  (0.06)  0.14 |
| L Banks of Superior Temporal Sulcus | 0.15  (0.17)  0.17 | -0.06  (0.02)  <0.001 | -0.01  (0.02)  0.79 | -0.11  (0.08)  0.14 |
| R Medial Superior frontal | 0.17  (0.09)  0.002 | -0.02  (0.01)  <0.001 | 0.001  (0.01)  0.95 | -0.02  (0.05)  0.62 |
| R Insula | 0.04  (0.12)  <0.001 | -0.03  (0.01)  <0.001 | -0.03  (0.02)  0.05 | 0.07  (0.06)  0.27 |

**Table S3:** Regression coefficients from a post-hoc LME analysis comparing CT developmental trajectories between CHEU and CHU in the 6 regions where children with PHIV demonstrated thicker cortex from age 5 to 9 years than HIV- controls. Numbering of regression coefficients is as per equation (2).

| Cluster region | HIV exposure status effects | Age effects | HIV exposure status by age interactions | Sex effects |
| --- | --- | --- | --- | --- |
|  |  |  |  |  |
|  | β_3_  (SE)  *p*-value | β_2_  (SE)  *p*-value | β_4_  (SE)  *p*-value | β_5_  (SE)  *p*-value |
| L Supramarginal | 0.16  (0.16)  0.62 | -0.02  (0.01)  0.003 | -0.02  (0.02)  0.37 | 0.03  (0.09)  0.69 |
| L Insula | 0.06  (0.24)  0.26 | -0.03  (0.02)  0.01 | -0.02  (0.03)  0.46 | -0.17  (0.09)  0.06 |
| L Lateral orbitofrontal | 0.11  (0.13)  0.85 | -0.03  (0.01)  <0.001 | -0.02  (0.02)  0.27 | 0.03  (0.07)  0.69 |
| L Banks of Superior Temporal Sulcus | -0.10  (0.23)  0.94 | -0.05  (0.02)  <0.001 | 0.01  (0.03)  0.65 | 0.01  (0.09)  0.90 |
| R Medial Superior frontal | 0.13  (0.11)  0.70 | -0.02  (0.01)  <0.001 | -0.02  (0.01)  0.26 | -0.05  (0.06)  0.39 |
| R Insula | 0.06  (0.16)  0.33 | -0.05  (0.01)  <0.001 | 0.002  (0.02)  0.91 | -0.03  (0.07)  0.70 |

**Table S4:** Regression coefficients from a post-hoc LME analysis comparing CT developmental trajectories between CPHIV in whom treatment was interrupted and those on continuous ART in the 6 regions where children with PHIV demonstrated thicker cortex from age 5 to 9 years than HIV- controls. Numbering of regression coefficients is as per equation (2).

| Cluster region | ART interruption status effects | Age effects | ART interruption status by age interactions | Sex effects |
| --- | --- | --- | --- | --- |
|  | β_3_  (SE)  *p*-value | β_2_  (SE)  *p*-value | β_4_  (SE)  *p*-value | β_5_  (SE)  *p*-value |
| L Supramarginal | -0.22  (0.14)  0.08 | -0.05 (0.01)  <0.001 | 0.01  (0.12)  0.53 | -0.16 (0.08) 0.03 |
| L Insula | 0.29  (0.23)  0.66 | -0.05 (0.02) <0.001 | -0.04  (0.03)  0.23 | -0.10 (0.10) 0.31 |
| L Lateral orbitofrontal | -0.13  (0.14)  0.63 | -0.05 (0.01) <0.001 | 0.01  (0.02)  0.42 | 0.09 (0.06) 0.13 |
| L Banks of Superior Temporal Sulcus | -0.11  (0.23)  0.18 | -0.06 (0.02) <0.001 | 0.002 (0.03)  0.95 | -0.11 (0.09) 0.22 |
| R Medial Superior frontal | 0.02  (0.11)  0.48 | -0.02 (0.01); 0.002 | -0.01  (0.02)  0.54 | -0.05 (0.06) 0.33 |
| R Insula | -0.05  (0.16)  0.44 | -0.03 (0.01) <0.001 | <-0.001 (0.02)  0.98 | 0.07 (0.07) 0.32 |


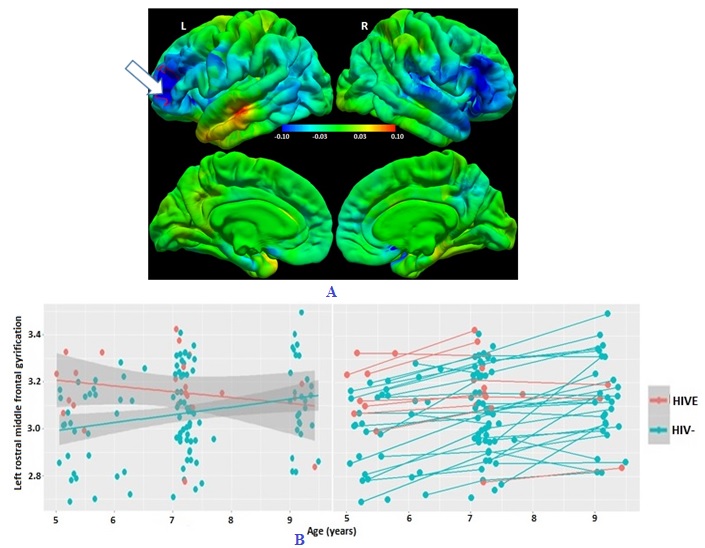


**Figure Caption**

**Figure S1**: Effects of HIV status (HIVE vs HIV-) by age interactions on local gyrification indices (LGIs). (A) Colour map of effects sizes. Positive regression coefficients (red/yellow) indicate greater rates of change of LGI in children who had previously been diagnosed with HIVE than HIV-, and negative coefficients (cyan/blue) indicate lower rates of change in HIVE than HIV- children. The colour bar scale applies to both lateral (top) and medial (bottom) views. Children with a previous diagnosis of HIVE did not demonstrate the increasing gyrification from ages 5 to 9 years evident in HIV- controls in bilateral rostral middle frontal regions. In the left hemisphere in the region outlined in red (white arrow), the difference is significant at an FDR-corrected *p*<0.05. (B) Plots showing the rate of change of gyrification in the left rostral middle frontal regions from ages 5 and 9 across HIVE and HIV- groups (left) and a spaghetti plot of individual trajectories (right). (HIV- = 66, HIVE = 12).
